# Editorial: Strengthening Child and Adolescent Mental Health (CAMH) Services and Systems in Lower-and-Middle-Income Countries (LMICs)

**DOI:** 10.3389/fpsyt.2021.645073

**Published:** 2021-02-05

**Authors:** Manasi Kumar, Amritha Bhat, Jürgen Unützer, Shekhar Saxena

**Affiliations:** ^1^Department of Psychiatry, University of Nairobi, Nairobi, Kenya; ^2^Department of Psychiatry and Behavioral Sciences, University of Washington, Seattle, WA, United States; ^3^Department of Global Health and Population, Harvard T.H. Chan School of Public Health, Harvard University, Cambridge, MA, United States

**Keywords:** child and adolescent mental health, health systems strengthening, community based interventions, screening, school based intervention and prevention

## Relevance of this Special Topic Today

More than half of all individuals living with mental illnesses present with their initial symptoms during childhood and adolescence (1). As more attention is paid to mental health in general, there is a growing realization that child and adolescent mental health in particular is inadequately understood and developed as a field. This is a global gap although the lack of support and service structures in low -and middle-income countries (LMICs) make it more challenging to assess the true burden. This lack of understanding of the true burden of child and adolescent mental health needs means it is more difficult to gather resources to develop systems and services to address this burden (2). This special edition aims to highlight the mental health needs of children and adolescents in LMICs by focusing on multilevel challenges, and on interventions and services, both novel and time-tested. A life course perspective in which assessment of child and adolescent development and psychosocial disturbances is integrated within existing systems can strengthen the entire health care system. This is pertinent for high income as well as low to middle income country settings. We decided to focus on LMICs as that is where most children and adolescents live and where there is the most need for CAMH professionals, services and system structures.

Since we published the final paper in this special topic, the SARS- COV-19 pandemic has taken the world by storm. Mental health services which were already stretched thin in most parts of the world have been critically impacted by the pandemic (3). Lockdowns and protracted closure of educational institutions, curfews preventing mobility of children and youth and high rates of financial instability due to job and business losses, illnesses and deaths due to COVID-19 infections, all led to increased rates of stress and poor mental health among children and adolescents (4). We hope these fifteen papers published between 2019 and 2020 will provide greater momentum to support CAMH, provide guidance on evidence based solutions to CAMH challenges, and help prioritize the well-being of children and youth during the pandemic and beyond.

## Overview of the Special Topic

We accepted 15 articles from diverse country contexts including China, Ecuador, India, Kenya, Malawi, Nigeria, Pakistan, Sierra Leone, South Africa, and Uganda. We included two systematic reviews on CAMH interventions and policies and one scoping review on use of technology for CAMH. The papers covered a wide range of themes from child development in the context of maternal depression (De Oliveira et al. from Pakistan), child and adolescent mental health system and services at national and provincial levels (Mokatimi et al. from South Africa), emotional and behavioral problems in children living with HIV and the concordance of these with their caregivers (van dan Heuvel et al. from Uganda), maternal nutrition and offspring stress response (Krishnaveni and Srinivasan from India), youth mental health policy in Sub-Saharan Africa (Kutcher et al. covering Tanzania and Malawi), impact of poor socioeconomic status on development of psychopathology (Perez-Mafril from Equador), CAMH services in post-emergency context (from van dan Brink Sierra Leone), differences between adolescent and adult perinatal depression (Nigeria Oladije et al.), HIV disclosure and stigma related factors in adolescents living with HIV (Nabunya Nabunuya et al. from Uganda), and a task-shared trauma intervention targeting children (Dorsey et al. in Kenya).

We included two policy focused papers, including one using a sustainability science and action lens to look at adolescent mental health, and a contribution on development of a WHO led caregiver skills program targeting children with developmental disabilities, delays and disorders (Salomone et al.). Aside from these we published three scoping and systematic reviews: CAMH policy lessons and gaps across LMICs (Zhou et al.), the potential of digital mental health to bolster child well-being (Huang et al.) and psychotherapy in LMICs for depression, trauma and anxiety disorders in children and adolescents (Uppendahl et al.). A reflective policy-practice paper addressed urban sustainability and youth mental health (Murphy et al.).

### Identification of Evidence-Based Interventions and Programs

These papers, and the exchanges we had with a number of pioneering researchers make it clear that there are many evidence-based approaches that can be integrated into heath care programming and service development in LMIC. Examples include using components of caregiver skills modules to support children with developmental disabilities (Salomone et al.) or trauma focused therapy delivered by lay community workers (Dorsey et al.), and the WHO led CST intervention which was designed to bridge a gap in addressing the needs of a highly vulnerable group of children with developmental disabilities and disorders.

### Potential for Scaling Up

There is a critical need to scale up well-tested interventions and intervention strategies like task-sharing and task-shifting in LMICs. Lay and non-specialist health workers can be trained to provide screening and structured psychosocial interventions. Such activities can be carried out at community, school, college, and primary care levels in various LMIC contexts to capture diverse settings and resources that may be available (Oladije et al., De Oliviera et al.).

## Challenges for the Field

### Increase Investment in CAMH

We hope through this special topic to increase attention to the severe underinvestment in this field and how this is impacting the well-being of children, youth and families across the world. Investment is needed to improve financing, but also for comprehensive economic costing. We need to explore and embrace the financial and opportunity cost of intervening and setting up requisite, responsive systems and services for children, adolescents, and families in need.

### Local Health, Community and Educational Capacity Building in Increasing Awareness and Toward Prevention and Promotion of Mental Health

The COVID-19 pandemic allows for opportunities to “build back better.” Techniques to build resilience, parenting skills and problem solving, and strategies to reduce stigma should be developed in community, health as well as educational contexts so that children, adolescents and their caregivers receive necessary support to bolster mental and behavioral health. Involvement of sectors other than health is critical for development of robust mental health promotion and prevention programs and policies. Reflective pragmatic and well-structured research in CAMH has a critical role here in shaping and bolstering the evidence.

Several papers point to improved assessment and measurement tools for capturing child and adolescent psychopathology. Adaptations and modifications of measurement tools to offer more contextualized assessment of child and adolescent distress and psychopathology is critical. Developing policies and National level programs that support such measurement, early screening and intervention approaches continue to be important. Early interventions targeting pregnant women, interventions around maternal and parental mental health are key to making a timely impact.

### Re-envisioning CAMH for LMICs During COVID-19

All countries of the world have been thrown into chaos with the rising numbers of infections, deaths and how families and communities are being impacted by the pandemic. Closures of learning institutions, care facilities for children and youth in need, and reduced possibilities of social networking, recreation and leisurely activities have impacted children and youth across the world. Children and adolescents in challenging situations such as children of migrants, internally displaced refugees, as well as families living in conflict, war or extreme poverty are particularly disenfranchised in this pandemic.

The motto of “building back better” in the context of mental health—has dual connotations—making missing CAMH structures in LMICs more robust, and bolstering services that are critically needed for recovery from the COVID-19 pandemic and beyond.

As can be seen from these papers risks such as poor nutrition, parental well-being, poverty and HIV remain critical issues for CAMH in LMICs. In such settings, the development of comprehensive and robust approaches that address social determinants through multimodal interventions and delivery platforms and a pragmatic and eclectic approach to intervention delivery may be particularly critical. Innovative strategies (Krishnaveni et al.) offered unique insight into maternal nutrition improvement to augment mental health and child well-being, Murphy et al. recommend the strategic framing and analysis of urban environment to address youth mental health, deep learning from colonial historical past to build back better mental health services in war and epidemic impacted regions—were recommendations from Mokatimi et al. and van dan Brink.

Approaches taken to strengthen policies and programs to improve child and adolescent mental health should be structured in developmentally appropriate ways, addressing barriers to mental health care and continually evaluating their effects on access to and outcomes of such services.

This vital scholarship has strengthened our conviction that service development, systems strengthening, and integration of effective mental health and substance use services in the broader context of health care need urgent prioritization in LMICs where the vast majority of children, adolescents, and families in need have little to no access to evidence-based care.

## Research Priorities Going Forward

Developing analytic tools to assess the long term impacts of COVID-19 on CAMH populations is especially important. For example, Christakis et al. (5) found that the decision to close US public primary schools in the early months of 2020 may be associated with a decrease in life expectancy for US children and associate this to over 5.53 million years lost. This estimate is for one of the highest income countries of the world. We do need such appraisals for countries badly impacted by COVID- such as Brazil, China, India, Columbia and also for those countries undergoing humanitarian and economic crises such as Yemen, Syria, Iraq, Afghanistan, Bolivia, etc. A special focus on countries that have poor to on CAMH structures, services or policies will be important.

There is a need for low cost and robust implementation strategies to reduce treatment gap, especially in very low resource environments. We need a research tested and accredited set of implementation tools which would enable uptake of low intensity interventions and strategies that would improve mental health outcomes of children, youth and families. Most implementation research has emerged from high income countries and tends to be focused on simplistic frameworks that do not capture the complexity of the LMIC structures or of multilevel social determinants in tandem. These health services and implementation models need to be piloted and advanced in LMICs.

CAMH friendly approaches to mental health promotion and resilience building is another pathway toward building positive competencies and skills that would enhance well-being. WHO/UNICEF nurturing care (7) and Lancet commission on child and adolescent mental health (6) have emphasized well-being by focusing on the future of children—this would necessitate action in terms of investment in early childhood, targeting prevention of commercial exploitation of children and youth, addressing climate change, poverty-reduction, social justice, right to education and health and promoting gender, racial and equity in keeping with the Sustainable Development Goals. Resilience building measures, life skills coaching and a capabilities framework were some suggestions toward developing a wholesome appraisal of well-being.

## Author Contributions

MK and SS drafted the outline. JU and AB edited. All authors read, revised, and approved the paper.

## Conflict of Interest

The authors declare that the research was conducted in the absence of any commercial or financial relationships that could be construed as a potential conflict of interest.
